# Future of total hip arthroplasty with the Metha short stem in modern surgeries

**DOI:** 10.1038/s41598-021-01367-3

**Published:** 2021-11-05

**Authors:** Marek Drobniewski, Marek Synder, Marek Aleksander Synder, Magdalena Krasińska, Lukasz Olewnik, Andrzej Borowski

**Affiliations:** 1 Orthopaedics and Paediatric Orthopaedics Department, Medical University of Lodz, ul. Pomorska 251, 92-213 Lodz, Poland; 2 Department of Anatomical Dissection and Donation, Medical University of Lodz, Lodz, Poland

**Keywords:** Outcomes research, Musculoskeletal system, Orthopaedics

## Abstract

The aim of the study was to analyse the results of uncemented total hip replacement, using the Metha (metaphyseal) stem. A total of 158 patients (70 females and 88 males) were qualified to the study and submitted to total hip arthroplasty (183 procedures altogether), using the Metha stem. The mean age of the patients on the day of surgery was 51.7 years (the range from 17 to 69 years). The mean follow up period was 9.2 years (the range from 5 to 13.5 years). Preoperative assessments gave poor scores, according to the Merle d’Aubigne and Postel classification, modified by Charnley. The average improvement after surgery, according to the used scale, was 6.9 points. A very good outcome was recorded in 154 cases (84.2%), a good outcome was achieved in 20 cases (10.9%) and a poor outcome was confirmed in 9 cases, while no satisfactory case was observed. Poor outcomes were associated with implant loosening. Extraskeletal ossification was noted in 10 cases (5.5%). According to the Kaplan–Meier estimator, the 10-year survival was 93.2% and 97.3% for the whole implant and the stem alone, respectively. 1. Our follow-up period of more than 9 years on the average, indicates that Metha stems produce excellent clinical and functional results in operated young patients with advanced degenerative changes of the hip joint. 2. Assuming a proper qualification for the procedure, the absence of complications and a correct surgical technique, which is slightly more difficult, when compared to standard stem implantation, the risk of aseptic loosening is fairly negligible.

## Introduction

Total hip joint arthroplasty (THA) has now become a common method of surgical intervention, applied in advanced forms of hip joint degenerative disease, while the indications for the application of this surgical method are growing in their number year by year. Endoprostheses are increasingly being used in difficult and complex cases of secondary coxarthrosis and in younger and younger patients. Multidirectional and diverse studies on implants, carried out by a broad spectrum of specialists, have given rise to the development of special implants which can be used, efficiently meeting their role, even in the most advanced hip joint deformations. Both acetabular components, with their diameters below 44 mm, and anatomical stems, with diameters of less than 10 mm, as well as the use of computer navigation and, eventually, the modularity of implants, make it possible to carry out arthroplasty surgery in actual hip joint conditions which may be highly deviated from the normal human hip joint layout^1–3^.

The multi-directional research and multilayered approach of specialists from various fields of science, including, among others, metallurgy, biomechanics and tribology, have the result that the orthopaedic surgeon, involved in the installation of hip joint implants, gets hold of increasingly more modern and durable implants. The actual development of endoprosthetics leads to an improvement of currently used implants, while offering more and more new engineering concepts for implant structure, shape and alloy composition, as well as for articulation and surgical procedure techniques. All these ongoing efforts are intended to design a hip implant with optimal biofunctional performance, structural strength and the longest possible service life in the patient’s body^4^.

In 2009, we published our initial experiences from implanting a new type of the Metha^®^ stem (BBraun/Aesculap), applied in hip arthroplasty procedures^5^ and, in 2015, we assessed, via densitometric examinations, the stem healing process into bone stock^6^. The very good functional, clinical and radiological results, obtained with Metha stems, prompted us to carry on the studies and evaluate hip joint arthroplasty results, while using the Metha stem over longer time periods and on larger groups of patients.

## Objectives of the study

The aim of the study was to analyse the long term results of uncemented total hip arthroplasty, using the Metha (metaphyseal) stem.

## Materials and methods

We performed retrospective study among all patients, who were operated in our hospital with Metha stem. We started using the Metha modular stem in June 2007. In a vast majority of the procedures, a Plasmacup acetabulum (BBraun/Aesculap) or, less commonly, a Screwcup acetabulum (BBraun/Aesculap) was used as the acetabular component. Although we had not recorded any complications with the modular stem (Isodur F Cobalt-Chromium forged alloy, CoCr29mo/iso 5832-12) prior to 2010, we then abandoned the use of those stems and switched to the Metha (metaphyseal) stem, in its monoblock version, which we have since then been implanting—(Metha Mono stem) with the inclination angle of 135°. The described stem is made of a forged titanium alloy, TiAl6V4, with the brand name of ISOTAN^®^F. Pure titanium is deposited in the proximal $${\raise0.5ex\hbox{$\scriptstyle 2$} \kern-0.1em/\kern-0.15em \lower0.25ex\hbox{$\scriptstyle 3$}}$$ of length with a 20 µm layer of dicalcium phosphate dihydrate (CaHPO4 × 2H2O), with the commercial name of Plasmapore µ-CaP. The sizes of implants vary by 1.5 mm and 1.3 mm in anteroposterior and axial projections, respectively. The difference in length between the smallest stem (size 0) and the largest one (size 7) is only 2.5 cm. At least a 5-year observation period was required to qualify patients to the reported study.

All the analysed surgical procedures were performed under epidural anaesthesia, each time from the anterolateral access without osteotomy of the greater trochanter. In all cases plastic templates were used during the preoperative planning. Attempts were made to install the acetabular component of the hip joint implant in the safe zone, as defined by Lewinnek^6^. As rule, the anteversion of the artificial acetabulum did not exceed 15° in our patients, while the stem was implanted in a small antetorsion of 5°–10°. The artificial acetabulum was seated exactly at the true acetabular region (TAR), i.e. where the original, natural acetabulum of the hip joint was localised. In the majority of cases, the metal shell of the acetabular component was completed with an asymmetric acetabular insert, made of polyethylene. In the youngest patients, operated before their 30th year of life, the use of ceramic heads of 32 mm in diameter is a standard at our Department. The rest of patients received same diameter, metal heads.

Antibacterial and antithrombotic prophylactic measures were implemented in the perioperative period. Improving exercises were recommended already on the postoperative day. After a Redon drain was removed, upright standing and learning to walk again exercises were started with feigned or full loading of the operated limb, depending on pain tolerance. Further intensive improving exercises were added on subsequent days.

Our study was retrospective in character. All the patients, included in the study, were clinically and radiologically examined before the planned arthroplasty and during the last outpatient follow up appointment in 2019. The patients were followed up after 3, 6 and 12 months from hospital discharge date; later on, subsequent follow up appointments were arranged with once a year frequency.

The obtained results of the clinical study were processed acc. to the classification of Merle d’Aubigne and Postel in Charnley’s modification. In this method, the following issues are subject to scoring: pain, walking ability and the sum of passive movement scopes within the operated hip joint^7^. Pain evaluation was done by means of the 10-point Visual Analogue Scale (VAS)^8^.

The Kellgren–Lawrence classification was applied for the assessment of preoperative radiograms^9^. Radiological imaging was also an integral part of follow up examinations. In each case, X-ray images of the operated hip joint were obtained in anteroposterior and axial projections. The radiograms were evaluated with respect to: hip implant positioning, with regards to both the artificial acetabulum and the stem, the degree of healing of the implant into the surrounding osseous tissue and the possible presence and degree of extraskeletal ossification^10,11^. In addition, possible migrations of the acetabular component were reviewed: horizontal, vertical and angular. The DeLee and Charnley classification system was used for the evaluation of acetabular component^12^, while the Gruen and Moreland classification was applied to evaluate the femoral stem. The axial seat of the stem in the proximal femoral epiphysis, features of vertical migration, bone atrophy, hypertrophy and saturation, plus the occurrence of intraosseous and periosteal ossification in seven zones were also assessed^13–15^. It is worth emphasizing that, in all the cases, radiological evaluations were carried out by an independent investigator (M.K.), not physically involved in the analysed surgical procedure.

The obtained results served as input for statistical analysis. Using the STATISTICA 10.0 PL program, the implant survival rate was calculated, using the Kaplan–Meier method with 95% Coefficient Intervals (95% Cl)^16^.

Informed consent was waived by Bio ethical Committee of the Medical University in Lodz, ref. No. RNN/178/14/KE.

All the procedures, carried out within the study project, were conformable with the ethical standard of the Helsinki Declaration in its version from 2013. There were approved by Bio ethical Committee of the Medical University in Lodz, ref. No. RNN/178/14/KE.

## Results

The study group definitively consisted of 158 patients, who underwent 183 hip arthroplasty surgeries with the METHA stem between the years 2007 and 2015. A total of 88 men (55.7%) and 70 women were treated. The right hip joint was operated on in 93 cases (50.8%) and the left hip joint was operated on in 90 cases. The mean age of the patients on the day of surgery was 51.7 years. (SD 9.8821; Me = 55). The youngest patient was 17 and the oldest one was 69 years old. The mean follow up period was 3 350.1 days (9.2 years), in the time range from 1837 days (5.0 years) to 4949 days (13.5 years), (SD 922.64; Me = 3370).

In addition, 16 extra arthroplasty operations on the opposite hip joint were carried out in the studied group of patients, using a Metha stem but, due to the follow-up period below 5 years, the results of those procedures were not further evaluated. The Plasmacup acetabular component was used in 173 procedures (94.53%) and the Screwcup acetabular component was used in the remaining 10 cases only. The size 3 Metha stem was the most commonly used femoral endoprosthetic component, implanted in 62 cases and representing 33.88% of all the analysed arthroplasty procedures. There were 54 modular stems (29.5%) and 129 monobloc stems (70.5%). In our series we used mainly the 135-degree neck, because during surgery it was the best position of the implant.

In the vast majority of cases (146 hip joints—79.8%), arthroplasty was indicated by idiopathic coxarthrosis. In 23 cases—12.6%, the indication for surgery was avascular necrosis of the femoral head (AVN), and the remaining 14 procedures concerned the cases of first-degree dysplastic coxarthrosis, according to the Crowe et al. classification. None of the patients had previously undergone surgery for hip disease. See Table 1 for a detailed characteristic of the study group with regards to selected parameters, with a division of the patients by gender.

**Table 1:** Characteristic features of the study group, divided by gender.

	Women	Men	Total number
The number of patients in the group	70 (44.3%)	88 (55.7%)	158
The number of operations	84 (45.9%)	99 (54.1%)	183
Right hip joint	48 (51.6%)	45 (48.4%)	93
Left hip joint	36 (40%)	54 (60%)	90
Two-stage procedure—left and right hip	14 (56%)	11 (44%)	25
The mean age of the patients on the day of surgery	51.3 years	52.0 years	51.7 years
Min.	20 years	17 years	17 years
Max.	69 years	69 years	69 years
The mean follow up period	3401 days (9.3 years)	3306.9 days (9.1 years)	3350.1 days (9.2 years)
Min.	1837 days (5 years)	1884 days (5.2 years)	1837 days (5 years)
Max.	4949 days (13.5 years)	4949 days (13.5 years)	4949 days (13.5 years)

After a mean follow-up period of 9.2 years from surgery, the following final results were obtained, using the modified Merle d’Aubigne and Postel classification. A very good outcome was recorded in 154 cases (84.2%), a good outcome was achieved in 20 cases (10.9%) and a poor outcome was confirmed in 9 cases (4.9%). No satisfactory outcome was obtained in any of the analysed cases. Using the Merle d’Aubigne classification, the average improvement was 6.9 points (p < 0.05). Nine (9) revision procedures were performed in the study group of patients. See Table 2 for detailed characteristics of revision surgery details.

**Table 2:** Revision surgery both for acetabular and femoral failure.

No.	Patient	Sex	Age	OA etiology	Side	Primary surgery date	Revision surgery day	Period of biofunctionality (days)	Type of the implant that was revised	Type of the implant that was re-implanted
1	K. R	M	53	Idiopathic	Right	16.06.2008	14.09.2016	3012	Insert PLASMACUP	Insert PLASMACUP
2	W. N	M	55	AVN	Left	27.07.2009	25.05.2011	667	Stem METHA	Stem ANTEGA
3	M. P	F	55	Idiopathic	Left	06.08.2009	07.08.2019	3653	Acetabular cup SCREWCUP	Acetabular cup PE-Cup
4	W. N	M	56	AVN	Right	24.02.2010	10.10.2012	959	Stem METHA	Stem ANTEGA
5	K. K	F	64	Idiopathic	Left	30.06.2010	04.09.2019	3353	Insert PLASMACUP	Insert PLASMACUP
6	L. S	F	53	Idiopathic	Right	05.04.2013	15.04.2013	10	Acetabular cup PLASMACUP	Acetabular cup PLASMACUP
7	K. R	M	58	Idiopathic	Right	08.10.2013	16.08.2018	1773	Acetabular cup PLASMACUP	Insert PLASMACUP
8	A. S	F	42	DDH	Left	04.08.2014	07.11.2016	826	Acetabular cup SCREWCUP	Acetabular cup PLASMAFIT
9	D. G	KF	48	DDH	Right	01.12.2014	24.04.2009	2047	Acetabular cup PLASMACUP	Acetabular cup AVANTAGE

Data containing patients: gender, age, OA etiology, date of revision, type of the implant that was revised.

*OA* osteoarthritis.

In seven cases, the realoplasty involved the acetabular component and only two procedures included Metha stem replacement. The reason for stem revision was, in either case, aseptic stem loosening, most probably due to the primary implantation of an undersized stem and, as it concerned some of the first implantations of the presented stem, it was then regarded as a learning curve. In addition, femoral nerve paresis was observed in two cases, with remission after 4 and 6 months from the surgery. No deaths were noted in the study group as well as neither septic nor thromboembolic complications. See Table 3 for detailed characteristics of the results, obtained in the study group, considering the division into the origin of degenerative changes.

**Table 3:** Clinical results by the modified Merle d’Aubigne and Postel's (MAP) grading, with consideration of the aetiology of degenerative changes.

Aetiology	MAP outstanding	MAP good	MAP satisfactory	MAP poor
Idiopathic (146 hips)	126	11	0	9
AVN (23 hips)	20	3	0	0
DDH (14 hips)	8	6	0	0
	154 (84.2%)	20 (10.9%)	0 (0%)	9 (4.9%)

*AVN* avascular necrosis of the femoral head, *DDH* developmental dysplasia of the hip.

Radiological evaluation, apart from the revision cases, as discussed above, showed no signs of aseptic endoprosthesis loosening, while confirming a correct and stable seat of the femoral component of the endoprosthesis in 173 cases (94.5%). The valgus position of the femoral component was noted in six cases, however, with no radiographic evidence of loosening. In addition, the presence of extraskeletal ossification was reported in ten cases (5.5%), which were classified as grade 1 and grade 2 in eight and two cases, respectively, according to Brooker’s division, which, however, had no negative impact on the quality of life of the operated patients.

Using the VAS scale, another research tool, we obtained the following results, the average score before surgery was 6.7, while the average score after hip arthroplasty was 1.2. The obtained score improvement was statistically significant (p < 0.05).

The subjective, post-operative evaluation of the surgery by the patients was much better from the final outcomes, achieved in the modified classification of Merle d’Aubigne and Postel. The biggest improvement was perceived in considerably reduced or entirely eliminated pain and in the increased mobility scope of the operated hip. The improvement of those parameters significantly contributed to an improved total, functional evaluation of the operated hip with a high level of patient satisfaction with the achieved outcome. Following our prognoses, the relatively worst final results were noted in the group of patients, operated for dysplastic coxarthrosis. On the other hand, analysing the above-mentioned results, one should keep in mind that the very good score, acc. to the modified Merle d’Aubigne and Postel classification, resulted from THA, the outcomes of which corresponded to healthy hip parameters. No thigh pain cases were found, while thigh pains are sometimes observed after uncemented hip arthroplasty.

Based on the obtained results, the probability of “survival” of the applied implants was determined, using the Kaplan–Meier estimator. The five-year probability of biofunctionality of the whole endoprosthesis was 95.1%, while amounting to 98.9% for the stem alone. Regarding a follow-up period of ten years, it was possible to determine the “survival” of the implants for 74 hip joints. The ten-year probability of biofunctionality was 93.2% and 97.3% for the whole implant and the stem alone, respectively. See Table 4 for the detailed results of biofunctional probability calculations.

**Table 4:** Kaplan–Meier biofunctionality coefficients for installed implants after 5 and 10 years from the surgery.

	Kaplan–Meier coefficients after 5 years (183 hips)	Kaplan–Meier coefficients after 10 years (74 hips)
The entire implant	95.0819% (91.8688–98.2950)	93.2432% (87.3206–99.1658)
The cup	96.1748% (93–3411-99.0085)	95.9459% (91.3583–100.5335)
The Metha stem	98.9071% (97.3924–100.4217)	97.2972% (93.5515–101.0430)

## Discussion

Total hip arthroplasty is a commonly used surgical method to treat advanced degenerative changes of the hip joint. While the choice of implant does not pose a great problem in elderly patients and is usually determined by the quality of bone tissue in which the implant is to be installed, it seems much more complex in younger people whose expectations of and hopes for improved quality of life are much higher than those of elderly patients.

The so-called unexplained thigh pain, most likely associated with too tight stem fitting in the femoral medullary canal, and the different elasticity levels of bone tissue and stem metal structures may be perceived as the few disadvantages of the uncemented implantation technique with standard stems. It concerns mainly the cases of the proximal end shape of the femoral bone of type A, according to Dorr^17^. Therefore, efforts have been undertaken to design a stem which could avoid such problems. In addition, it was decided to apply a coating of bioactive materials on stem surface, the role of which is to prevent the release of metal ions, increase the osteointegration process of the implant with bone tissue and avoid any unwanted penetration of the fibrous membrane into the space between the stem and the bone stock.

Research conducted in this direction has brought about new designs. One of such solutions was the concept of short-stemmed implants, known as metaphyseal stems. In its assumptions, the implantation of a metaphyseal stem presents many advantages, the major ones of which include the small implant size, the coating with an active substance that promotes secondary stability, the possibility of using a minimally invasive technique and the possible modularity of the implant, which enables a more accurate restoration of correct biomechanical conditions in the operated joint (offset). The higher, i.e. more bone-sparing femoral neck osteotomy is not insignificant, either for the future of artificial joints, as it lets preserve more bone stock in operated hip joint. As originally intended, the implantation of a metaphyseal stem enables more physiological load transfers and, owing to the implant design, the risk of unexplained, post-arthroplasty thigh pains is eliminated. A comparison of the biomechanical features between the classical, anatomical stem and the metaphyseal stem showed, in the latter case, higher physiological load transfers at the femoral neck region and beneficial effects for osteointegration and bone remodelling at this region, preventing the adverse effect of *stress shielding*^18^.

Our many-year experience with Metha stems in our patients confirms the reports of other authors about the high effectiveness of these implants in the treatment of hip degenerative joint disease. Already our first results, admittedly still with short follow-up periods, reflected in the high satisfaction of operated patients and, in addition, in densitometric examinations of Meta stem penetration into bone stock, have strengthened our conviction, regarding the high clinical values of this solution focused technique. Those aspects of our earlier study prompted us to carry on the research and evaluate the results of arthroplasty with Meta stems over longer follow-up periods and in larger groups of operated patients.

Our results are fully conformable with literature reports.

Malahias et al. reviewed the results of 12 scientific papers on hip arthroplasty procedures using Metha stems. The study group comprised a total of 5048 patients, aged between 44.4 and 60.4 years. During a follow-up period of 2 to 9 years, the rate of implant-related revision was determined to be 2.5% and the rate of major complications, however not demanding revision surgery, was 2.8%^19^.

In turn, Schnurr et al. reported on 1734 hip arthroplasties, using three types of Metha stems (1090 Metha Mono stems, 314 modular stems with a titanium adapter and 230 modular stems with a cobalt-chromium adapter). During a mean follow-up period of 6 years, 15 (4%) modular stem adapter failures were reported. The 7-year revision rate for Metha Mono stems was found to be 1.5%. The revision rate was 1.8% and 5.3% for modular stems with a cobalt-chromium adapter and a titanium adapter, respectively^20^.

In another study, von Lewinski et al. analysed the results of 1953 hip arthroplasty procedures, using Metha stems. There were 38 revision procedures in the study group. In 12 cases, the reason for endoprosthesis replacement was a damaged titanium stem adapter, in 19 cases, implant loosening, in 5 cases, periprosthetic fractures and, in 2 cases, femoral medullary canal perforation. The revision rate was 1.9%. Standard cementless stems were used for revision procedures in 34 cases, in two cases, metaphyseal stems were implanted and only two cases demanded revision implants. Neither implant dislocation nor thigh pains were reported after hip arthroplasty^21^.

Thorey et al. analysed the results of 151 hip arthroplasty surgeries with Metha stems. The mean age of operated patients was 55.7 years and the mean follow-up period was 5.8 years. The Harris Hip Score and HOOS (Hip Osteoarthritis Outcome Score) classifications were employed for clinical assessment. The mean clinical score prior to surgery was 46 and increased to 90 after operation. The mean HOOS clinical scores were 55 and 89 before and after the surgery, respectively. There were only two revision demanding cases of stem sinking and one case of septic complication which, however, did not require revision of the femoral component. The Kaplan–Meier coefficient was 98%^22^.

Also, Wittenberg et al. presented the results of 250 hip arthroplasty procedures with METHA stems. The mean age of operated patients and the mean follow-up period were 4.9 years and 60 years, respectively. The mean HOOS clinical scores were 50 and 97 before and after the surgery, respectively. The VAS scale was used for pain assessments. The VAS results were 7.4 and 0.23 before and after surgery, respectively. Nine revision procedures were required for the following reasons: aseptic stem loosening (3 cases), infection (3 cases) and femoral medullary canal perforation. Further nine revisions were demanded for titanium stem adapter failures. After 5 years, the Kaplan–Meier coefficient was 96.7%^23^.

In our opinion, the success of Metha stem implantations is associated with the precise and strict adherence to the medical indications to surgery. In our study material, we initially qualified only primary degenerative changes of the hip joint for the surgery. Only after about 5 years of follow-up did we extend our indications to cases of sterile necrosis of the femoral head and mild forms of dysplastic coxarthrosis (grade I according to the Crowe et al. classification). We also avoided using Metha stems in cases of severe valgus or varus deformity of the operated hip joint and when bone quality seemed poor. Any previous surgical interventions on the proximal end of the femoral bone provided an automatic contraindication to using this type of stem in our material. In our opinion, confirmed by densitometric studies, the absolute prerequisite for the use of a Metha stem is a good quality of the proximal femoral bone tissue.

From 2010 onwards, there is an increasing number of articles reporting dual modular stem design prosthesis failure, where sudden fractures of the modular neck at the neck-stem junction were reported as early as first years after implantation^24–26^. The reasons for a modular neck fracture of a Ti alloy are multifactorial^26^. Failure of modular titanium alloy neck adapters can be initiated by surface micromotions due to surface contamination or highly loaded implant components^26^.

Atwood et al. reported on fractures of modular titanium (Ti) alloy necks coupled with Ti alloy stems. Fretting at a modular junction continually wears away the passive oxide layer that provides Ti6Al4V alloy implants with protection from corrosion, requiring constant repassivation. Repassivation in a crevice depletes the limited oxygen supply, thus decreasing the local pH and promoting further corrosion, resulting in pitting and the formation of sharp cracks^27^. In our series we did not observe such complication, however modular stem should be used with extreme caution especially in patients with an average weight over 100 kg^26^.

In our material, dysplastic coxarthrosis represented only 7.6% of the analysed cases and those were hip joints classified as type I, according to the Crowe et al. classification, which had not been surgically treated for that reason before. However, studies by other authors confirmed the efficacy of this stem also in more advanced forms of dysplastic coxarthrosis.

Suksathien et al. analysed the results of 32 hip arthroplasty operations with Metha stems, performed in patients with dysplastic coxarthrosis of type I and II, according to the Crowe et al. classification. The mean age of operated patients and the mean follow-up period were 50.3 years and 7 years, respectively. Neither loosening of any implant component nor joint dislocation or peripheral nerve injury was noted. In their conclusions, the authors underscore the promising clinical results in the medium-term follow-up period and emphasise the physiological load transfer through the proximal end of the femur^28^.

Budde et al. also evaluated the results of hip arthroplasty with a Metha stem, applied in cases of dysplastic coxarthrosis. The study group consisted of 58 patients and the follow-up period was 2.9 ± 1.1 years. The authors also put a strong accent in the conclusions on the possibility of adequate restoration of normal biomechanical conditions in the operated hip joint and the obtained good clinical results^29^.

Another indication to use Metha stems is hip arthroplasty to treat sterile necrosis of the femoral bone head. In our material, 12.6% of arthroplasty procedures were performed in patients with AVN of various aetiology.

Suksathien et al. analysed the results of 83 hip arthroplasty operations, using Metha stems, indicated for sterile necrosis of the femoral head. The mean age of operated patients was 43.8 years and the mean follow-up period was 69.3 years. Using the HHS scale, the mean improvement was reflected in the rise from 44.7 points at baseline to 99.6 points at the final postoperative assessment. Four cases of intraoperative femoral bone fractures and one case of stable perforation of the distal femoral medullary canal in its distal section were observed in the study group of patients. One revision procedure was performed for a peri-prosthetic fracture, four years after the endoprosthesis implantation, and one revision surgery was required by aseptic loosening of the acetabular component. After five years, the Kaplan–Meier coefficient was 98.8%^30^.

On the other hand, Merschin et al. presented an interesting comparison of the results of 52 hip arthroplasty surgeries with Metha stems, performed for sterile necrosis of the femoral head in 26 patients, with a control group. The Oxford Hip Score (OHS) was used for clinical assessment, achieving similar follow-up results in both groups. During a mean follow-up period of 65.88 months, there was one case of modular stem adapter fracture that required revision surgery. In the conclusions, the authors recognise the Metha stem implantation procedure as a valuable treatment method, indicated for sterile necrosis of the femoral head in young patients^31^.

In our material, 23 procedures (12.6%) of hip arthroplasty with Metha stem were performed for sterile necrosis of the femoral bone head. Our results are similar to those of the above-mentioned studies and fully confirm the reports of other authors^32–34^.

Many authors, pay attention to the precision and accuracy of surgical technique, while implanting the metaphyseal stem. Compared to standard stems, the Metha stem implantation technique is more demanding. The higher level of femoral neck resection itself results in a limited access and insight into the space of the operated joint, what may pose a barrier to successful implantation of the acetabular component. We used intraoperative X-ray control in each case during Metha stem implantation to assess the correct seating of implanted stem. We paid special attention to correct positioning of the stem, to make it rest against the lateral cortical layer of the femoral bone in the anteroposterior projection and firmly seated in the femoral neck axis in the axial projection. According to other authors, the use of computer navigation can also contribute to optimal stem placement^35^.

The DEXA (Dual Energy X-ray Absoptiometry) evaluation of bone tissue at the area of the operated joint, is a really interesting aspect of the studies on implant healing in process.

Augustin et al. reviewed the results of 43 hip arthroplasty procedures with Metha stems, indicated after densitometric assessment of bone remodelling around the hip stem. DEXA showed an initial reduction in bone mineral density at zones 1 and 7, followed by a regular increase in those values up to baseline over a longer follow-up period. We may thus conclude that force transmission and load distribution around the Metha stem are correct^36^.

In turn, Fisher et al. reported on a clinical evaluation and densitometric findings after 1 year from 48 hip arthroplasty surgeries with Metha stems. Densitometry showed an increase in bone mineral density in the proximal and distal lateral zones around the Metha stem and a decrease in bone mineral density in the proximal medial and central lateral zones around the tested implant. The reported observation confirms the process of remodelling and healing of the stem into the mataphyseal region of the proximal femoral bone^37^.

Other densitometric findings, confirming bone remodelling around the Metha stem and the implant integration into the surrounding bone tissue, were published by Falez, Yan, Synder, Brinkmann, Jahnke and Lerch et al.^6,38–42^.

Considering our clinical material, we believe that the Metha stem is a valuable and safe solution for patients with hip degenerative joint disease, demonstrating only a slight risk of complications.

## Conclusions

1. Our follow-up indicates that Metha stems produce excellent clinical and functional results in operated young patients with advanced degenerative changes of the hip joint.
2. Assuming a proper qualification for the procedure and a correct surgical technique the risk of aseptic loosening is fairly negligible.
